# A Tube-Integrated Painted Biosensor for Glucose and Lactate

**DOI:** 10.3390/s18051620

**Published:** 2018-05-18

**Authors:** Weihua Shi, Xiaojin Luo, Yue Cui

**Affiliations:** College of Engineering, Peking University, Beijing 100871, China; sswwhh@pku.edu.cn (W.S.); luoxiaojin@pku.edu.cn (X.L.)

**Keywords:** biosensor, painted, tube, enzyme, glucose, lactate, hydrogen peroxide

## Abstract

Developing a simple and direct approach for sensitive, specific, and rapid detection of metabolic compounds is of great importance for a variety of biological, medical, and food applications. Tubes are a highly portable and accessible container shape which are widely used for scientific research in cell biology and chemical synthesis, and which are also of great use in domestic health care applications. Here, we show for the first time the development of a tube-based painted amperometric biosensor for the detection of glucose and lactate. The sensor was prepared by printing carbon graphite and silver/silver chloride inks on the interior wall of the tube and then immobilizing glucose oxidase or lactate oxidase on the sensor. The sensor showed a sensitive, rapid, and reliable detection of glucose and lactate. We anticipate that these results could open new avenues for the development of painted biosensors, and toward advanced biosensor applications.

## 1. Introduction

The development of new types of bioelectronic sensors could expand opportunities for both fundamental new research and for a variety of applications in healthcare, defense, and environmental monitoring [1,2]. Conventional bioelectronic sensors are based on hard substrates, such as silicon substrates and ceramics [1,2]. Recently, new developments in non-conventional electronics have attracted great attention for their advantages in flexibility, wearability, and real-time or onsite monitoring [3,4]. Our recent prior research has demonstrated several new printed and painted bioelectronic sensors, such as a plastic film-based biosensor [5], a regular paper-based biosensor [6], a cotton fabric–based biosensor [7], etc.

Tubes are a highly portable and accessible container shape which are widely used for scientific research in cell biology [8,9] and chemical synthesis [10], and which are also of great use in domestic health care applications [11,12]. Tubes are used as containers for an extremely wide variety of solutions, including enzymes [12,13], toxic chemicals [14], cell cultures [15,16], urine and blood samples [17], saliva samples [18], etc. Equipping tubes with built-in sensors can simplify chemical detection procedures and provide real-time statuses of analyte concentrations.

Amperometric biosensors are miniaturized sensing devices that can provide rapid, sensitive, and reliable detection of target analytes [19,20]. Glucose is an important analyte for biomedical applications, such as diabetes monitoring and cell biology [21,22]. Lactate is another important biomedical indicator and can be detected in oral fluids or sweat [23,24], thus making it extremely suitable for building non-invasive biosensors. It is therefore highly desirable to develop amperometric biosensors for the detection of glucose and lactate that can be constructed in a small tube. The sensor templet that we developed in this research performed these tasks effectively, and holds promise for broader applications in other fields if other bioreceptors are immobilized.

In this work, we demonstrate for the first time the development of a tube-based painted amperometric biosensor for glucose and lactate. The sensor was constructed by printing carbon graphite and silver/silver chloride (Ag/AgCl) inks on the interior wall of the tube to construct working, reference, and counter electrodes. Glucose oxidase (GOD) or lactate oxidase (LOD) was then immobilized on the working electrode of the sensor. A potential was applied between the working electrode and reference electrode. When the sensor was in the presence of glucose/lactate, the glucose/lactate was catalyzed by GOD/LOD to generate hydrogen peroxide (H_2_O_2_), which was then oxidized by the working electrode to generate a current response.

## 2. Materials and Methods

### 2.1. Apparatus and Chemicals

A potentiostat CHI660e was purchased from CH Instruments, Inc. (Shanghai, China). An oven was purchased from YiHeng, Inc. (Shanghai, China). An optical microscope was purchased from Cewei, Inc. (Shanghai, China). Carbon graphite ink and Ag/AgCl ink were purchased from Gwent Electronics Materials LTD (Pontypool, UK). Foam tipped swabs were purchased from Chengjinghao Electronic Co., Ltd. (Suzhou, China). Silver paste was purchased from Nanjing Xylite, Inc. (Nanjing, China). Sodium phosphate monobasic dihydrate was purchased from Tianjin Fuchen Chemical Reagents Factory (Tianjin, China). Glucose anhydrous was purchased from Tianjin Zhiyuan Reagent Company, Ltd. (Tianjin, China). Glutaraldehyde, sodium L-lactate, and H_2_O_2_ were purchased from Sigma-Aldrich, Inc. (Beijing China). GOD and LOD were purchased from Toyobo Co., Ltd. (Osaka, Japan). Plastic tubes (volume: 2 mL, length: 42 mm, diameter: 11 mm) were purchased from Runing Medical Instruments Co., Ltd. (Taizhou, China).

### 2.2. Sensing Electrode Preparation

The tube-based sensor was prepared with three electrodes, including a working electrode, a reference electrode, and a counter electrode. The working and counter electrodes were painted with carbon graphite ink, and the reference electrode was painted with Ag/AgCl ink. All three electrodes were painted vertically on the interior wall of the tube using foam tipped swabs manually, and each electrode had a dimension of 35 mm in length and 5 mm in width. After printing, the electrodes were dried in an oven at 80 °C for 30 min, followed by cooling at room temperature. Silver paste was then added to the top ends of the electrodes to connect the painted electrodes to wires.

### 2.3. Enzyme Functionalization

For glucose sensors, diluted glutaraldehyde (2%) and GOD (10 U μL^−1^) were mixed at a volume ratio of 1:1. For lactate sensors, diluted glutaraldehyde (2%) and LOD (50 U μL^−1^) were mixed at the same volume ratio of 1:1. For each kind of sensor, 3 μL of the mixture was placed at the bottom end of the working electrode manually with an auto pipette, covering an area of 5 mm in width and 5 mm in length. The tube was then laid horizontally with the painted electrode facing down to dry at 4 °C overnight. On the following day, it was placed at room temperature and incubated with a buffer solution for about 1 h before measurement.

### 2.4. Sensing Measurements

The sensing measurement of the tube-based sensor was performed with the potentiostat at room temperature (27 °C). The three electrodes were connected to the potentiostat with wires. A constant potential of 0.6 V between the working electrode versus the Ag/AgCl was applied for the amperometric measurements. The tube was placed in a tube holder vertically during the whole measurement. A volume of 0.45 mL of the phosphate buffer (50 mM, pH 7.5) was placed in the tube, covering 0.75 mm of the bottom ends of these electrodes. When the current became stable, 50 μL of H_2_O_2_ or glucose/lactate with different concentrations were injected into the buffer sequentially. All solutions are injected manually with an auto pipette. Upon adding a certain concentration of analyte into the buffer, the current signal changed and stabilized rapidly. After this, another concentration of analyte was added into the buffer and the same results were achieved. The current-versus-time curves for the detection of H_2_O_2_ or glucose/lactate were recorded, and a calibration curve between the current response and the analyte concentration was also plotted. Cyclic voltammograms for these sensors were studied with a scan rate of 30, 50, 70, or 100 mV/s, in a buffer solution (50 mM, pH 7.5) containing 2 mM of H_2_O_2_, or glucose/lactate.

## 3. Results and Discussion

**Figure 1:**
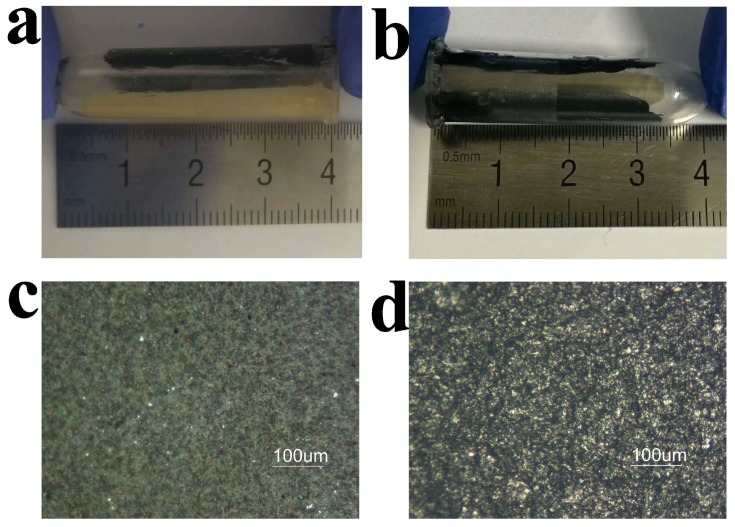
Images of a tube-based sensor. (**a**) A camera image of the sensor that shows a carbon graphite electrode and a Ag/AgCl electrode; (**b**) A camera image of the sensor that shows two carbon graphite electrodes; (**c**) An optical image of the Ag/AgCl electrode; (**d**) An optical image of the carbon graphite electrode.

Figure 1 shows the camera image and optical image of the tube-integrated sensor. The camera images (Figure 1a,b) show that the working, reference, and counter electrodes were successfully painted on the interior wall of the tube, for the construction of the amperometric biosensor. The electrodes are thin and straight. Figure 1b clearly indicates that the electrodes are discrete from each other. The black electrodes were painted with carbon graphite ink and functioned as the working and counter electrodes. The grey electrode was painted with Ag/AgCl ink and functioned as the reference electrode. The optical image shows that the Ag/AgCl electrode (Figure 1c) and carbon graphite electrode (Figure 1d) were uniformly painted on the wall surface. The tube was cut open to allow the picturing of these electrodes. By using a micrometer, the thicknesses of the electrodes were measured, the carbon graphite electrode has a thickness of 95 μm, and the Ag/AgCl electrode has a thickness of 260 μm. These results demonstrate that the sensor was successfully constructed on the curved inside surface of the tube.

**Figure 2:**
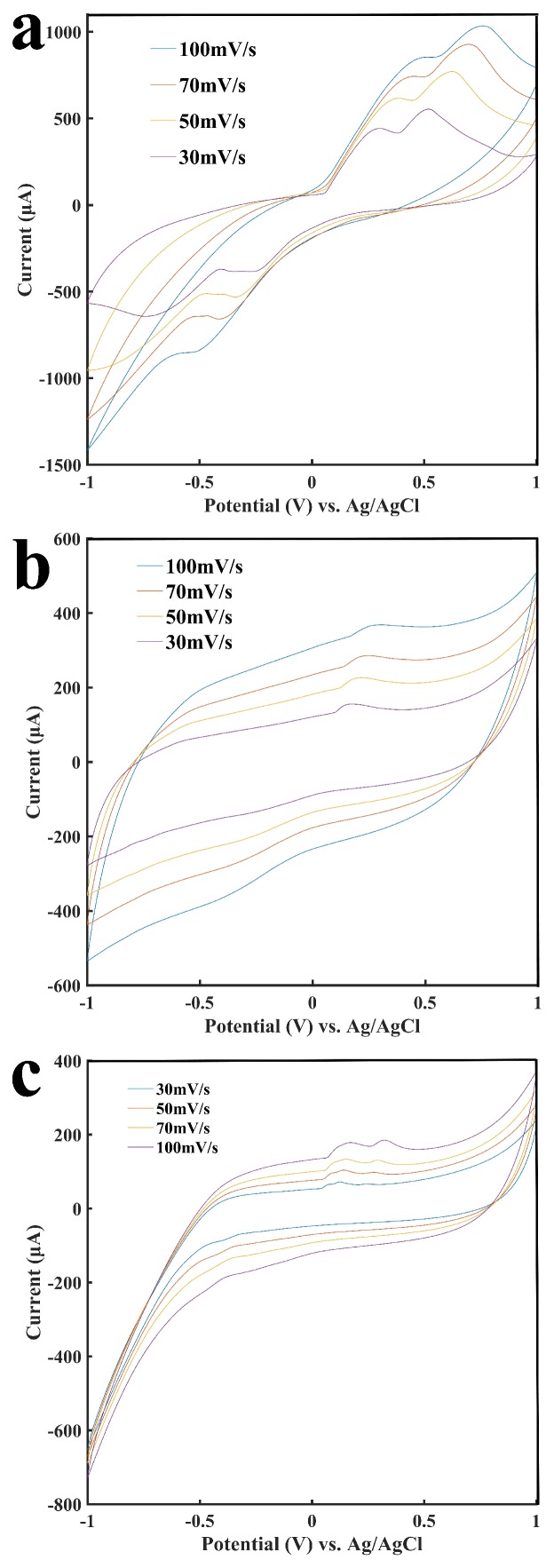
Cyclic voltammograms of the sensor. (**a**) Cyclic voltammogram of the sensor with 2 mM H_2_O_2;_ (**b**) Cyclic voltammogram of the glucose oxidase-immobilized sensor with 2 mM glucose. (**c**) Cyclic voltammogram of the lactate oxidase-immobilized sensor with 2 mM lactate.

Figure 2 shows the cyclic voltammograms of the sensors. Figure 2a shows the cyclic voltammogram of the sensor in a buffer solution containing 2 mM H_2_O_2_, which was involved in the oxidation-reduction reaction at the surface of the electrode at different potentials. Figure 2b shows the cyclic voltammograms of the enzyme-immobilized sensor in a buffer solution containing 2 mM glucose. Figure 2c shows the voltammograms of the enzyme-immobilized sensor in a buffer solution containing 2 mM lactate. Through the enzymatic reaction, glucose and lactate were catalyzed to generate H_2_O_2_, which was then detected by the electrode to generate a current response. When the scanning rate changed from 30 to 100 mV/s, the oxidation/reduction currents became slightly different due to different oxidation/reduction processes. A comparison of Figure 2a–c clearly demonstrates that glucose or lactate resulted in smaller current responses under the same potential as compared with the current response from H_2_O_2_. This was expected since H_2_O_2_ is directly involved in the oxidation and reduction on the electrode surface, while glucose or lactate is catalyzed by GOD or LOD, respectively, to produce H_2_O_2_ before the oxidation/reduction of H_2_O_2_. The shapes of the curves as shown in Figure 2 are related to the property of the painted electronic inks, and these curves show similar shapes. The curves also showed us that the electrode had a strong oxidation at a potential at 0.6 V; comparing the CV curves in the presence of analytes and in the absence of analytes, there are clearly differences of the current signals at 0.6 V (see Figure S9 in Supplementary Material). Therefore, we chose 0.6 V for subsequent amperometric measurements.

**Figure 3:**
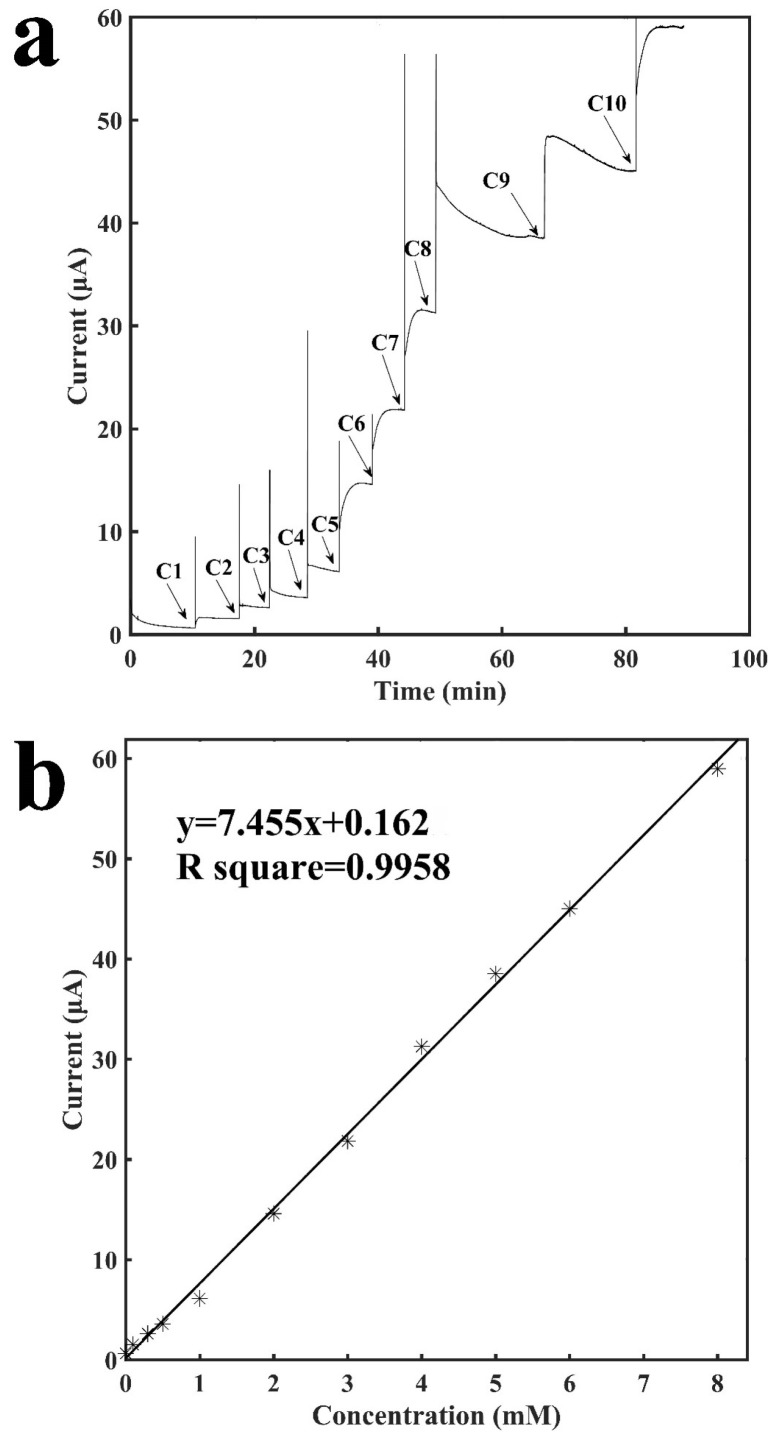
Characterization of the sensor for the detection of H_2_O_2_. (**a**) Current vs. time signal response curve to different concentrations of H_2_O_2_, C1: 0.1 mM, C2: 0.2 mM, C3: 0.2 mM. C4: 0.5 mM, C5: 1.0 mM, C6: 1.0 mM, C7: 1.0 mM, C8: 1.0 mM, C9: 1.0 mM, C10: 2.0 mM; (**b**) Calibration curve for the detection of H_2_O_2_.

Figure 3a shows the current-versus-time response curve of the tube-integrated sensor for detection of H_2_O_2_, which is the end product of a variety of oxidase-based enzymatic reactions. The buffer solution resulted in a stable current baseline, and when H_2_O_2_ was injected it diffused to reach to the surface of the electrode and was oxidized by the electrode to generate a current response. When the entire process reached stability, the current reached a new stable status. The sensor response to the injection of H_2_O_2_ was rapid and the entire measurement process usually took less than 5 min, and occasionally, it may be a little longer due to the diffusion issue since H_2_O_2_ was manually injected into the tube. Due to the injection of H_2_O_2_, the current response was directly proportional to the concentration of H_2_O_2_, and it increased as the concentration increased. The sensor also showed excellent repeatability. As shown from C5 to C9 in Figure 3a, the current response to the increase of 1 mM H_2_O_2_ was repeatable. Figure 3b shows the calibration curve for the detection of H_2_O_2_. It shows a linear relationship between the current response and the concentration of H_2_O_2_ ranging from 0.1 mM to 8 mM with a slope of 7.46 μA mM^−1^ and a R^2^ of 0.9958. Two other sensors were built to examine the reproducibility and they have: a slope of 7.38 μA mM^−1^ and a R^2^ of 0.9708; and a slope of 7.35 μA mM^−1^ and a R^2^ of 0.9903 (see Figures S1 and S2 in Supplementary Materials). The sensor had a detection limit of 12.8 μM (signal to noise ratio of 3). The results demonstrated the sensitive and rapid detection of H_2_O_2_ with a tube-based painted sensor, which indicates the possibility for construction of a variety of biosensors based on this sensor configuration via the immobilization of bioreceptors, such as enzymes, antibodies, etc.

**Figure 4:**
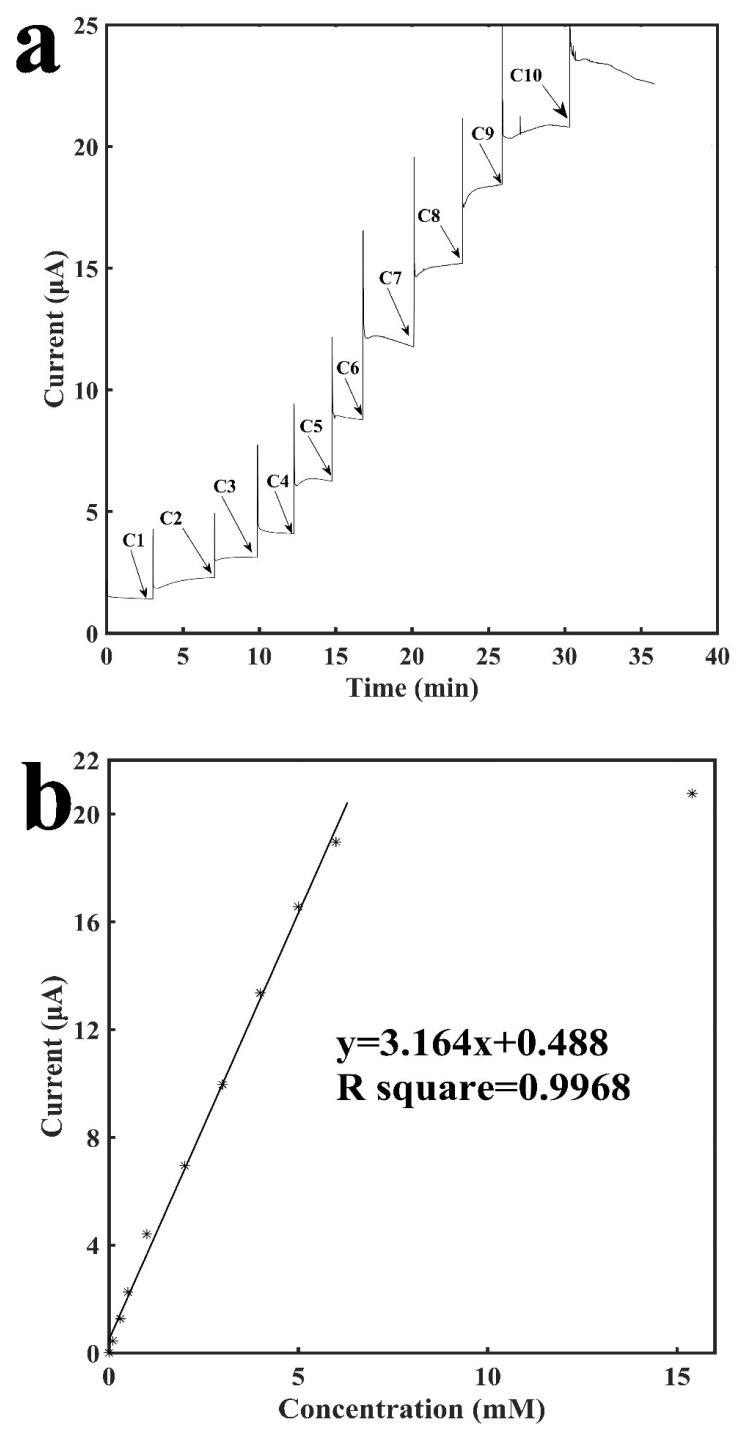
Characterization of the glucose oxidase-immobilized sensor for the detection of glucose. (**a**) Current vs. time signal response curve to different concentrations of glucose, C1: 0.1 mM, C2: 0.2 mM, C3: 0.2 mM. C4: 0.5 mM, C5: 1.0 mM, C6: 1.0 mM, C7: 1.0 mM, C8: 1.0 mM, C9: 1.0 mM, C10: 9.4 mM (**b**) Calibration curve for the detection of glucose.

Figure 4 shows the characterization of the tube-integrated biosensor with the immobilization of GOD for the detection of glucose. Glucose was catalyzed by GOD to generate H_2_O_2_ on the surface of the working electrode. Therefore, by detecting H_2_O_2_, which is the end product from the enzymatic reaction, glucose could be detected. Similarly, buffer was added into the tube with the sensing electrodes, and after the current baseline became stable, different concentrations of glucose were added into the tube to generate current increases. Figure 4a shows the current-versus-time response curve for detection of glucose. It shows that although the sensor took around 3–5 min for the current to finish stabilizing, usually its signal response was reached within half a minute with an error rate of no more than 10% as compared with the final current response. The current response increased as the concentration of glucose increased. The sensor also demonstrated excellent repeatability. As shown from C5 to C9 in Figure 4a, the current response to the increase of 1 mM glucose was repeatable. Figure 4b shows the calibration curve for the sensing of glucose. It shows a linear relationship between the current response and the concentration of glucose ranging from 0.1 mM to 6 mM with a slope of 3.16 μA mM^−1^ and a R^2^ of 0.9968. Three other GOD sensors were built to examine the reproducibility and they have: a slope of 3.13 μA mM^−1^ and a R^2^ of 0.9677; a slope of 2.61 μA mM^−1^ and a R^2^ of 0.9679; a slope of 3.06 μA mM^−1^ and a R^2^ of 0.9771 (see Figures S3–S5 in Supplementary Materials). At a higher concentration of 15.4 mM, the signal response is not linearly proportional to the concentration of glucose anymore, which may be due to the saturation of enzymatic reaction. The sensor has a detection limit of 16.5 μM (signal to noise ratio of 3).

**Figure 5:**
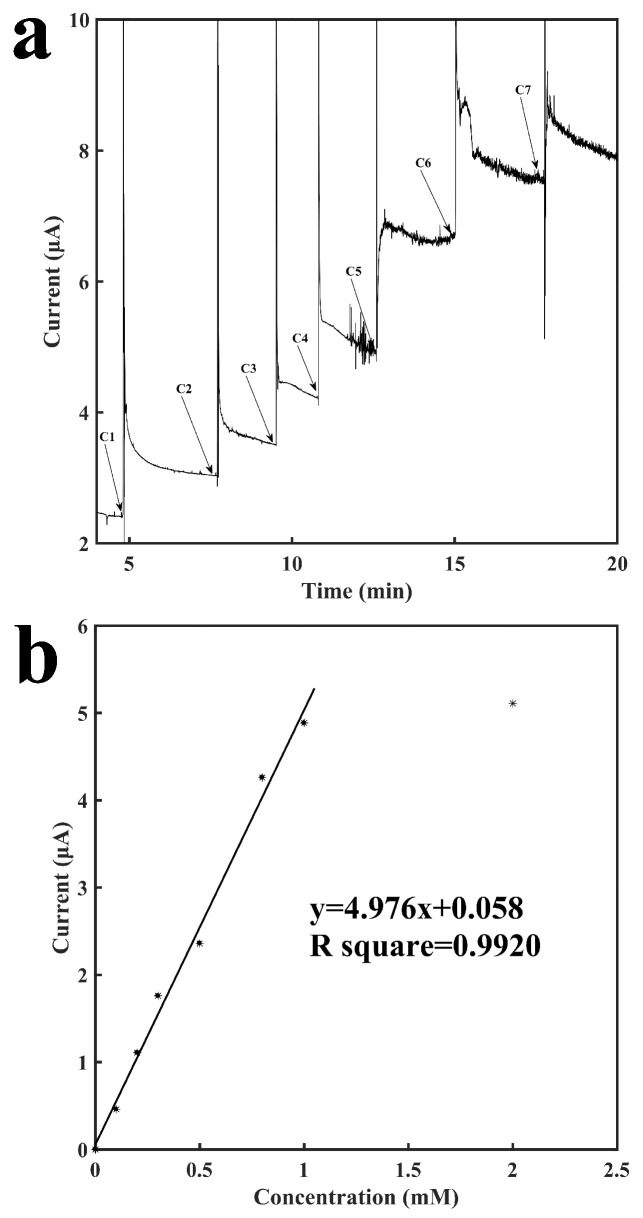
Characterization of the lactate oxidase-immobilized sensor for the detection of lactate. (**a**) Current vs. time signal response curve to different concentrations of lactate, C1: 0.1 mM, C2: 0.1 mM, C3: 0.1 mM. C4: 0.2 mM, C5: 0.3 mM, C6: 0.2 mM, C7: 1.0 mM (**b**) Calibration curve for the detection of lactate.

Figure 5 shows the characterization of this biosensor with the immobilization of LOD for the detection of lactate. Similar to the process described above for glucose, lactate is catalyzed by LOD to generate H_2_O_2_, which was detected on the surface of the working electrode. The addition of buffer solution and additional different concentrations of lactate solutions had the same results as the glucose tests described above. Figure 5a shows the current-versus-time response curve for detection of lactate. Similar to glucose, although the current needed around 3–5 min to finish stabilizing, the response time was usually less than 1 min with an error rate of no more than 10% as compared to the final result. As indicated in the graph, the current responses also increased as the concentration of lactate increased. The sensor showed an excellent repeatability as well. As shown from C1 to C3 in Figure 5a, the current response to the increase of 0.1 mM lactate was repeatable. Figure 5b shows the calibration curve for the sensing of lactate. It shows a linear relationship between the current response and the concentration of lactate ranging from 0.1 mM to 1 mM with a slope of 4.98 μA mM^−1^ and a R^2^ of 0.9920. Three other LOD sensors were built to examine the reproducibility and they have: a slope of 5.42 μA mM^−1^ and a R^2^ of 0.9402; a slope of 5.26 μA mM^−1^ and a R^2^ of 0.9302; a slope of 4.27 μA mM^−1^ and a R^2^ of 0.9565 (see Figures S6–S8 in Supplementary Materials). At a higher concentration of 2 mM, the signal response is no longer linearly proportional to the concentration of lactate, which may be due to the saturation of enzymatic reaction. Increasing the units of the immobilized enzymes could possibly extend the detection range of the sensor. In the mean time, the tube provides a volume that is large enough for samples to be diluted before measurement. Therefore, the relatively low saturation concentration should not affect the practical application for monitoring lactate concentration that is above 1 mM. The sensor had a detection limit of 84.8 μM (signal to noise ratio of 3).

These results demonstrate that the tube-based sensors we constructed can be used for sensitive and rapid detection of both glucose and lactate. Although stabilization times were longer at high concentrations for both glucose and lactate, overall detection times were short, and the longer stabilization times at high concentrations may have been caused by the longer process of the analyte’s diffusion. Fluctuations at high concentrations were not concerning since they well exceeded the detection ranges and therefore did not affect the calibration curve of the sensors. These fluctuations may have been caused by enzyme saturation, but further study is required before a final conclusion can be reached. The sensors could be applied for both one one-time measurement and continuous monitoring of glucose or lactate concentrations in various healthcare, molecular biology, or food applications. Overall, our results indicate the possibility of construction of a variety of other tube-integrated biosensors predicated on the immobilization of other bioreceptors.

## 4. Conclusions

In this work, we have shown for the first time the development of a tube-integrated glucose/lactate biosensor and its successful application for the sensitive and rapid detection of glucose/lactate. Our sensor construction method was simple, with carbon graphite and Ag/AgCl inks printing onto the interior wall of a tube and glucose/lactate oxidase immobilized on the sensor. The process was easy to perform, low cost, and rapid. The sensor showed a reliable detection of glucose/lactate with a broad detection range and a rapid measuring time. This approach may provide new avenues for constructing a variety of other tube-based sensors with applications in healthcare, molecular biology, defense, food safety, and environmental monitoring.

## Supplementary Materials

The following are available online at http://www.mdpi.com/1424-8220/18/5/1620/s1.
