# Supplementary material for: A Tube-Integrated Painted Biosensor for Glucose and Lactate
Source: Sensors (Basel). 2018 May 18;18(5):1620. doi: 10.3390/s18051620 (PMC5982665; doi:10.3390/s18051620)
Supplement: Supplementary file 1 [file sensors-18-01620-s001.zip › sensors-302046-supplementary.docx]

Supplementary Material for

Tube-Integrated Printed Biosensor for Glucose and Lactate

Weihua Shi, Xiaojin Luo and Yue Cui*

College of Engineering, Peking University, Beijing 100871, China

***** Correspondence: ycui@pku.edu.cn; Tel.: +86-10-82524860

Repetition for H_2_O_2_ sensors





**Figure S1.** Characterization of the sensor for the detection of H_2_O_2_. First repetition. (a) Current vs. time signal response curve to different concentrations of H_2_O_2_, C1: 0.1 mM, C2: 0.2 mM, C3: 0.2 mM. C4: 0.5 mM, C5: 1.0 mM, C6: 1.0 mM, C7: 1.0 mM, C8: 1.0 mM, C9: 1.0 mM (b) Calibration curve for the detection of H_2_O_2_.





**Figure S2.** Characterization of the sensor for the detection of H_2_O_2_. Second repetition. (a) Current vs. time signal response curve to different concentrations of H_2_O_2_, C1: 0.1 mM, C2: 0.2 mM, C3: 0.2 mM. C4: 0.5 mM, C5: 1.0 mM, C6: 1.0 mM, C7: 1.0 mM, C8: 1.0 mM, C9: 1.0 mM, C10: 2.0 mM; (b) Calibration curve for the detection of H_2_O_2_.

**Repetition for glucose sensors**





**Figure S3.** Characterization of the glucose oxidase-immobilized sensor for the detection of glucose. First repetition. (a) Current vs. time signal response curve to different concentrations of glucose, C1: 0.1 mM, C2: 0.2 mM, C3: 0.2 mM. C4: 0.5 mM, C5: 1.0 mM, C6: 1.0 mM, C7: 1.0 mM, C8: 1.0 mM, C9: 1.0 mM, C10: 9.4 mM (b) Calibration curve for the detection of glucose.





**Figure S4.** Characterization of the glucose oxidase-immobilized sensor for the detection of glucose. Second repetition. (a) Current vs. time signal response curve to different concentrations of glucose, C1: 0.1 mM, C2: 0.2 mM, C3: 0.2 mM. C4: 0.5 mM, C5: 1.0 mM, C6: 1.0 mM, C7: 1.0 mM, C8: 1.0 mM, C9: 1.0 mM, C10: 9.4 mM (b) Calibration curve for the detection of glucose.





**Figure S5.** Characterization of the glucose oxidase-immobilized sensor for the detection of glucose. Third repetition. (a) Current vs. time signal response curve to different concentrations of glucose, C1: 0.1 mM, C2: 0.2 mM, C3: 0.2 mM. C4: 0.5 mM, C5: 1.0 mM, C6: 1.0 mM, C7: 1.0 mM, C8: 1.0 mM, C9: 1.0 mM, C10: 9.4 mM (b) Calibration curve for the detection of glucose.

**Repetition for lactate sensors**





**Figure S6.** Characterization of the lactate oxidase-immobilized sensor for the detection of lactate. First repetition. (a) Current vs. time signal response curve to different concentrations of lactate, C1: 0.1 mM, C2: 0.1 mM, C3: 0.1 mM. C4: 0.2 mM, C5: 0.3 mM, C6: 0.2 mM, C7: 1.0 mM, C8: 1.0 mM (b) Calibration curve for the detection of lactate.





**Figure S7.** Characterization of the lactate oxidase-immobilized sensor for the detection of lactate. Second repetition. (a) Current vs. time signal response curve to different concentrations of lactate, C1: 0.1 mM, C2: 0.1 mM, C3: 0.1 mM. C4: 0.2 mM, C5: 0.3 mM, C6: 0.2 mM, C7: 1.0 mM, C8: 1.0 mM (b) Calibration curve for the detection of lactate.





**Figure S8.** Characterization of the lactate oxidase-immobilized sensor for the detection of lactate. Third repetition. (a) Current vs. time signal response curve to different concentrations of lactate, C1: 0.1 mM, C2: 0.1 mM, C3: 0.1 mM. C4: 0.2 mM, C5: 0.3 mM, C6: 0.2 mM, C7: 1.0 mM, C8: 1.0 mM, C9: 1.0 mM (b) Calibration curve for the detection of lactate.

**CVs in the absence of analytes for H_2_O_2_ /GOD /LOD sensors**





**Figure S9.** Cyclic voltammograms of the sensors in the absence of analytes. (a) Cyclic voltammogram of the sensor in buffer_;_ (b) Cyclic voltammogram of the glucose oxidase-immobilized sensor in buffer. (c) Cyclic voltammogram of the lactate oxidase-immobilized sensor in buffer.
